# Enhanced bone regeneration capability of chitosan sponge coated with TiO_2_ nanoparticles

**DOI:** 10.1016/j.btre.2019.e00350

**Published:** 2019-06-05

**Authors:** Radyum Ikono, Ni Li, Nanda Hendra Pratama, Agnia Vibriani, Diah Retno Yuniarni, Muhammad Luthfansyah, Boy Muchlis Bachtiar, Endang Winiati Bachtiar, Kamarza Mulia, Mohammad Nasikin, Hideaki Kagami, Xianqi Li, Etik Mardliyati, Nurul Taufiqu Rochman, Tokiko Nagamura-Inoue, Arinobu Tojo

**Affiliations:** a Division of Bionanotechnology, Nano Center Indonesia, Jl. Raya Serpong, 15310, Tangerang Selatan, Indonesia; b Department of Metallurgical Engineering, Sumbawa University of Technology, Jl. Raya Olat Maras, 84371, Nusa Tenggara Barat, Indonesia; c Division of Molecular Therapy, Institute of Medical Science, The University of Tokyo, 7 Chome-3-1 Hongo, 113-8654, Tokyo, Japan; d Department of Oral and Maxillofacial Surgery, Matsumoto Dental University, 1780 Hirookagobara, Shiojiri, Nagano-Prefecture, 399-0704, Japan; e Department of Biology, Bandung Institute of Technology, Jl. Ganesha No. 10, 40132, Bandung, Indonesia; f Department of Chemistry, University of Indonesia, Jl. Margonda Raya, 16424, Depok, Indonesia; g Oral Science Laboratory, Department of Dentistry, University of Indonesia, Jl. Salemba Raya, 10430, Central Jakarta, Indonesia; h Department of Chemical Engineering, University of Indonesia, Jl. Margonda Raya, 16424, Depok, Indonesia; i Center for Pharmaceutical and Medical Technology, Agency for the Assessment and Application of Technology (BPPT), PUSPIPTEK Area, 15314, Tangerang Selatan, Indonesia; j Research Center for Physics, Indonesian Institute of Science (LIPI), PUSPIPTEK Area, 15314, Tangerang Selatan, Indonesia; k Department of General Medicine, IMSUT Hospital, The Institute of Medical Science, The University of Tokyo, 7 Chome-3-1 Hongo, 113-8654, Tokyo, Japan; l Department of Cell Processing and Transfusion, The Institute of Medical Science, The University of Tokyo, 7 Chome-3-1 Hongo, 113-8654, Tokyo, Japan

**Keywords:** Bone regeneration, Chitosan, Sponges, TiO_2_ nanoparticles, Tissue engineering

## Abstract

Chitosan has been a popular option for tissue engineering, however exhibits limited function for bone regeneration due to its low mechanical robustness and non-osteogenic inductivity. Here we hybridized chitosan with TiO_2_ nanoparticles to improve its bone regeneration capability. Morphology and crystallographic analysis showed that TiO_2_ nanoparticles in anatase-type were distributed evenly on the surface of the chitosan sponges. Degradation test showed a significant effect of TiO_2_ nanoparticles addition in retaining its integrity. Biomineralization assay using simulated body fluid showed apatite formation in sponges surface as denoted by PO^4−^ band observed in FTIR results. qPCR analysis supported chitosan - TiO_2_ sponges in bone regeneration capability as indicated by DMP1 and OCN gene upregulation in TiO_2_ treated group. Finally, cytotoxicity analysis supported the fact that TiO_2_ nanoparticles added sponges were proved to be biocompatible. Results suggest that chitosan-50% TiO_2_ nanoparticles sponges could be a potential novel scaffold for bone tissue engineering.

## Highlights

• Chitosan hybridized with titanium dioxide nanoparticles improves its bone regeneration capability.
• Nano titanium dioxide addition to the matrix of chitosan sponges was done successfully, as depicted from an even distribution of nano titanium dioxide on the surface of the sponges.
• Chitosan – nanoTiO_2_ scaffold results in significantly improved sponge robustness, biomineralization, and bone regeneration capability, as indicated by DMP1 and OCN gene upregulation in chitosan-50% nanoTiO_2_ sample.

## 1 Introduction

Nano Titanium dioxide (TiO_2_) has been widely investigated for many applications, due to its unique properties exhibited in nano size [[1], [2], [3], [4]]. Some prominent applications due to its photocatalytic activity, self-cleaning surface ability, low toxic and others [[5], [6], [7], [8], [9], [10]]. Despite extensive investigations of nano TiO_2_ on diverse fields, its discussion on its potentials in tissue engineering field are still limited.

Several studies have shown that nano TiO_2_ could improve bioactivity and mechanical properties [39,40], when coated in a composite-like structure with, for instance bioglass, silk, and other polymers [[11], [12], [13], [14], [15], [16], [17], [18]]. Nevertheless, study on nano TiO_2_ coating to other type of biopolymers, also its possibility to induce osteogenesis has yet to be elucidated thoroughly.

In this study, we used chitosan that has long been used in many biomedical applications as sponge, hybridized with TiO_2_ nanoparticles. Chitosan has been regarded as a biocompatible carbohydrate polymers that have been tested widely for biomaterials application, including tissue regeneration [[19], [20], [21]]. In terms of oral tissues, chitosan has been proven to be able to act as a supporting matrix for regeneration of several soft oral tissues [[22], [23], [24], [25], [26]]. However, chitosan alone as a scaffold for regeneration has a limited function for bone formation [27].

At present, most commonly used scaffolds for bone tissue engineering are hard type biomaterials including hydroxyapatite and β-tricalcium phosphate. Although relatively soft and flexible texture of chitosan is beneficial and may fit better for complex shape cavities, several studies have highlighted that chitosan scaffold could easily be degraded in the body due to insufficient mechanical properties [28,29]. The insufficiency in mechanical properties also leads to inadequate capacity for bone formation [30]. Mooney et al, for instance, reported that the mechanical properties of the scaffold is strongly correlated to the type of tissue targeted to be regenerated; thus for hard tissue (e.g. bone, dental) regeneration, scaffold with higher strength is more preferable [31]. We hypothesized that coating with nano TiO_2_ will significantly improve its bioactivity, mechanical properties, and also osteogenic inductivity, thus promote accelerated bone formation. If that is the case, chitosan-nano TiO_2_ system could be useful to regenerate bone in uncommon shape cavities, which are commonly observed for tooth extraction socket, periodontal tissue defect and the cavities after tumour or cyst removal.

Previous study by Kumar (2018) showed that chitosan scaffold doped by nano TiO_2_ resulting improvement on physiochemical properties. Nevertheless, its biological activity, especially on bone formation capability, as well as osteoinductivity was yet to be examined. This research main focus is to investigate chitosan-nano TiO_2_ biological activity, specifically on its bone formation and osteogenesis supporting capability.

## 2 Materials and methods

All procedures of experiments using animals in this study were performed in accordance with “The guidelines laid down by the National Institute of Health (NIH) in the USA” regarding the care and use of animals for experimental procedures and approved by “Matsumoto Dental University Committee on Intramural Animal Use” (No. 289).

### 2.1 Fabrication of chitosan-nano TiO_2_ hybrid sponge

The chitosan-nano TiO_2_ sponge (CTS) was prepared by dissolving 1 g of chitosan powder (Sigma Aldrich) and nano TiO_2_ with particle size 20 nm (Degussa p25, purity: 99.9%) (wt% of TiO_2_:chitosan was set at 1:2, 1:4, 1:8) into 50 ml of 1% (v/v) CH_3_COOH (Merck) in the glass beaker to yield solution A. The solution A was then stirred vigorously for 1 h to homogenize the solution. The solution of NaOH (Merck) was prepared by diluting 2 g of NaOH with 50 ml of deionized water in the glass beaker, yielding solution B. CTS was synthesized by adding solution A into solution B at room temperature, stirred until precipitate was formed, then separated from the solution using paper filter. The CTS was then placed into a 96-well culture plate (as a mold) and was frozen overnight at -30^0^C. The frozen CTS was then lyophilized, then subsequently washed in the deionized water two times to obtain final CTS.

### 2.2 Surface morphology observation of the sponges

Scanning electron microscope (SEM) was used to study the surface morphology of the CTS. The analysis was carried out using a SEM (FEI Quanta 650) with an Oxford INCA/ENERGY-350 microanalysis system.

### 2.3 Crystallographic analysis of the sponges

To confirm the crystal structure of nano TiO_2_ after addition to chitosan solution, X-Ray diffraction analysis was carried out using XRD diffractometer (XRD, RIGAKU, RINT 2100/PC) with a *CuK*_α_ (λ =1.5406 Å)

### 2.4 Biomineralization activity of the sponges

CTS with equal weight was immersed in 1x simulated body fluid (SBF) solution and incubated in closed tube at 37^0^C for 1 and 2 weeks. After that, CTS was washed three times using deionized water to remove adsorbed mineral, and appearance after treatment was captured by digital camera. Chemical analysis of the samples were performed by FTIR spectroscopy. Experiment was conducted in triplicate.

### 2.5 Preparation of mouse mesenchymal stem cells (MSCs)

Male C57BL/6 J mice (3 weeks old, SLC Japan, Hamamatsu, Japan) were sacrificed with overdose anesthesia. The femurs and tibiae were disconnected from the trunk and soft tissues were removed from the bone surface thoroughly. Epiphyses were cut and bone marrow was flushed out using a syringe and #27 needle with a culture medium consisted with α-minimum essential medium with glutamine and phenol red (α-MEM, Wako Pure Chemical Industries, Ltd., Osaka, Japan) supplemented with 1% penicillin-streptomycin-amphotericin B solution (Biological Industries Israel Beit Haemek Ltd., Kibbutz Beit Haemek, Israel). The mononuclear cells were separated in order to obtain pure cells homogenate, using density gradient centrifugation (Lymphoprep™, Cosmo Bio Co. Ltd., Tokyo, Japan) as indicated by the manufacturer’s protocol. The isolated mononuclear cells were washed twice and seeded on a culture dish (Falcon®, Corning, USA) at the density of 5.5 × 10^5^/cm^2^ in the basic culture medium consisted with α-MEM supplemented with 10% FBS, 1% penicillin-streptomycin-amphotericin solution and 10 ng/ml recombinant human basic-fibroblast growth factor (**b-**FGF; Pepro Tech, Rocky Hill, NJ, USA). The primary cells were cultured at 37 ℃ in a 5% CO_2_ humidified incubator. Medium was changed every three days. When the cells reached 70–80% confluence, the cells were detached with 0.25% trypsin-EDTA (Gibco: Life Technologies, Carlsbad, CA, USA) and subcultured in a new culture dish at a density of 1.5 × 10^4^ cells/cm^2^ until subconfluent. The cells at second passage were used in this study.

### 2.6 Osteogenic induction of MSCs on the sponges

All sponges were sterilized with UV irradiation for 30 min. When the MSCs reached 50–60% confluence, 1 × 10^4^ cells/cm^2^ was cultured on the sponges of different nano TiO_2_ concentration (0%, 12.5%, 25%, and 50%). The basic culture medium was then replaced with osteogenic induction medium (basic culture medium supplemented with 100 nM dexamethasone (Sigma-Aldrich, St. Louis, MO, USA), 50 μM L-ascorbate acid phosphate (Wako Pure Chemical Industries, Ltd.) and 10 mM glycerol phosphate disodium salt hydrate (Sigma-Aldrich Co. LLC.). During the induction process, the media were changed every two days.

### 2.7 RNA extraction and quantitative real-time RT-PCR (qRT-PCR)

qRT-PCR was performed to determine the expression of osteogenic markers (OCN gene and DMP1 gene). Briefly, total RNA was extracted using TRIzol reagent (Ambion®; Life Technologies, Carlsbad, CA, USA). After quantification of total RNA with a spectrophotometer (Nano Drop® ND-1000, Thermo Fisher Scientific, Waltham, MA, USA), RNA samples were reverse-transcribed into complementary DNA (cDNA) using oligo (dT)12–18 primers (Life Technologies), dNTPs (Toyobo Co. Ltd, Osaka, Japan) and ReverTra Ace® (Toyobo Co., Ltd.) according to the manufacturer’s instructions. qRT-PCR were performed in a thermal cycler (Thermal Cycler Dice Real Time System II TP-900, Takara Bio, Japan) using SYBR Premix Ex TaqII reagent (Takara Bio, Kusatsu, Japan) according to the manufacturer’s protocol. Primer sets (Sigma-Aldrich Co.) used for the PCR experiment were listed in Table 1.

**Table 1:** qRT-PCR primer sets.

Primer	Direction	Sequence (5’-3’)
***β-Actin***	forward	CATCCGTAAAGACCTCTATGCCAAC
	reverse	ATGGAGCCACCGATCCACA
**DMP1**	forward	AGTGAGTCATCAGAAGAAAGTCAAGC
	reverse	CTATACTGGCCTCTGTCGTAGCC
**Ocn**	forward	CTCTGTCTCTGACCTCACAG
	reverse	GGAGCTGCTGTGACATCCATAC

### 2.8 Cytotoxicity analysis

For the cytotoxicity analysis, mouse bone marrow MSCs were prepared as described above. MSCs (2.5 × 10^5^) were seeded on each scaffold (30 mg, approximately 0.8 × 0.8 x 0.8 mm) and the cell number was measured at day 5 after seeding. Less cell number correspond to higher cytotoxicity of the CTS. Cell number were evaluated with a WST-8 assay (Cell Counting Kit-8, Dojindo Laboratories, Kumamoto, Japan) according to the manufacturer’s instructions. The value of Cell Counting Kit-8 was measured at 450 nm in a multi-detection microplate reader POWERSCAN® HT (Dainippon Pharmaceutical, Osaka, Japan).

### 2.9 Visualization of attached cells on the scaffold

Mouse bone marrow MSCs were seeded on the scaffold and cultured for 24 h. Then the cell-scaffold complexes were fixed with 4% formalin, washed in PBS, and incubated with 0.2% crystal **violet** (Wako) at room temperature for 5 min. After washing with distilled water, the scaffold with cells was put on a dish, cover slipped and observed under a stereoscopic microscope (LEICA MZ6, Leica Microsystems)

### 2.10 Statistical analysis

The results were presented as means ± standard error of means. Statistical analyses were conducted using Student’s *t*-test between two groups. The P-value of less than 0.05 was considered statistically significant.

## 3 Results

**Fig. 1:**
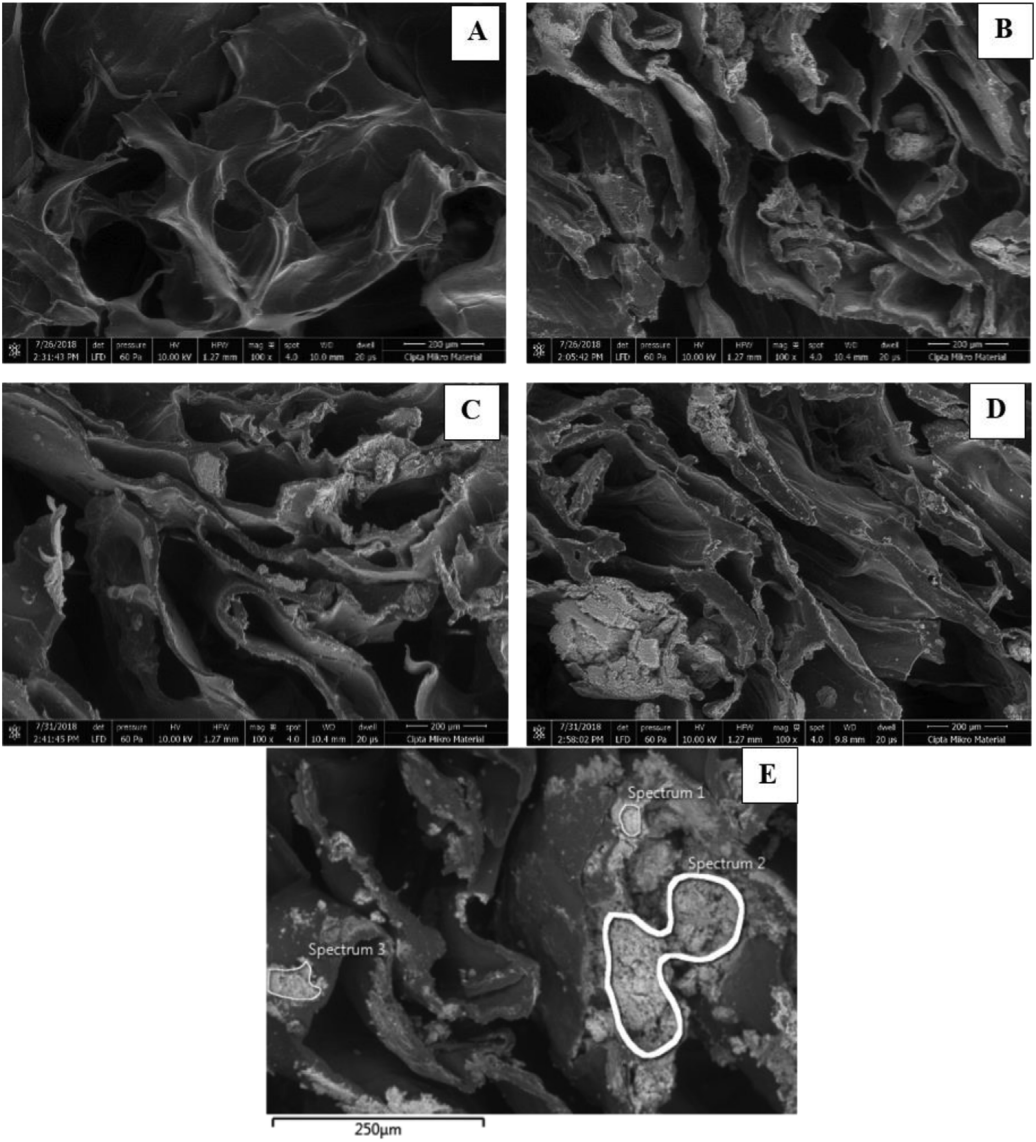
SEM images of chitosan (chi) - nano TiO_2_ (NT) sponge of different treatment group: (A) control, (B) chi-12.5%NT, (C) chi-25%NT, and (D) chi-50%NT (E) SEM – EDS image of chi-12.5%NT sponge.

Chitosan – nano TiO_2_ sponges were successfully fabricated. Fig. 1 shows the morphology of the CTS at 100x magnitude using SEM.

All sponges showed significant number of pores and interconnectivity, as standard requirements of good scaffold in tissue engineering application. TiO_2_ nanoparticles were spread relatively evenly in all samples added with TiO_2_ nanoparticles. Qualitatively, increasing concentration led to more TiO_2_ nanoparticles attached to the surface of the chitosan sponges, as depicted in Fig. 1D that agglomeration of TiO_2_ was observed in some parts of the sponge. The TiO_2_ addition was also confirmed quantitatively by EDS analysis, as shown in Table 2, apparent concentration value of Ti and O element has the highest value compared to other elements.

**Table 2:** Quantitative Results of EDS Analysis of chi-12.5% NT sponge.

Element	Spectrum 1			Spectrum 2			Spectrum 3		
	Wt (%)	Atomic(%)	Apparent Conc.	Wt (%)	Atomic (%)	Apparent Conc.	Wt (%)	Atomic (%)	Apparent Conc.
**C**	6.44	12.23	5.06	6.94	14.78	3.77	8.09	15.42	5.35
**O**	44.63	63.59	45.50	32.65	52.21	19.00	41.59	59.54	34.26
**Na**	1.75	1.73	2.89	1.26	1.41	1.45	1.91	1.90	2.67
**Ti**	47.19	22.46	61.79	59.15	31.60	55.15	48.41	23.14	53.30
**Total:**	100.0	100.0		100.0	100.0		100.0	100.0	

**Fig. 2:**
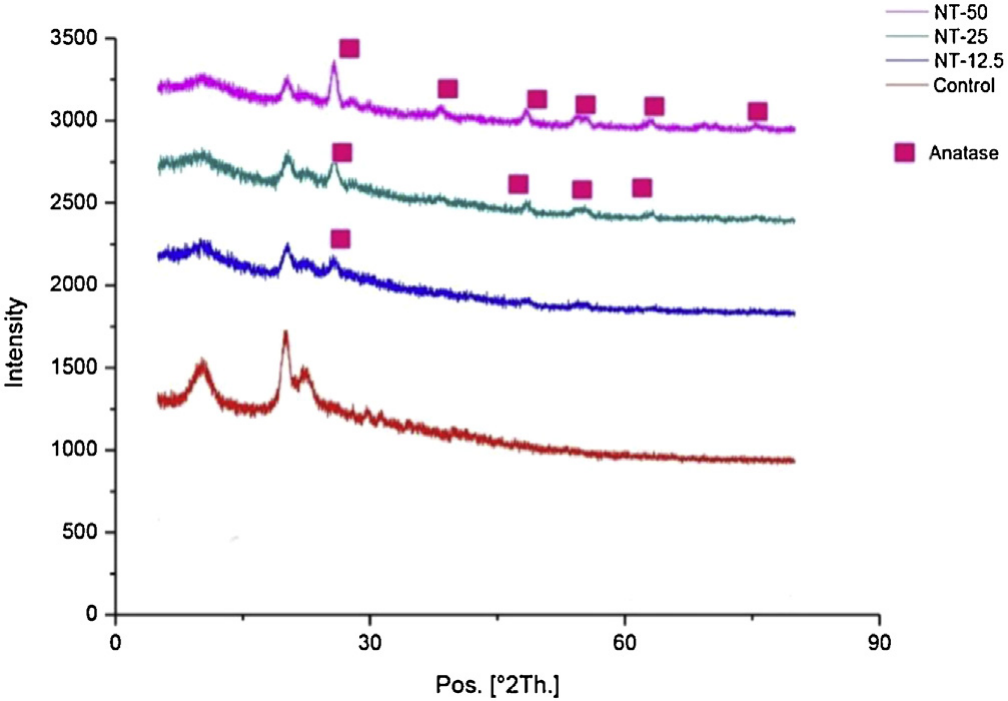
XRD analysis of different sample groups. This graph shows that addition of TiO_2_ to the sponge did not affect the crystal structure of TiO_2_ shown by anatase crystal structure retention. (NT—X : nano TiO_2_ – concentration used).

XRD analysis was further carried out to confirm the existence, also the crystal structure of TiO_2_ after sponge synthesis. It can be inferred from Fig. 2 that TiO_2_ existed in the sponge with increasing pattern, as increasing TiO_2_ concentration. It was further confirmed that TiO_2_ maintained its anatase crystal structure.

**Fig. 3:**
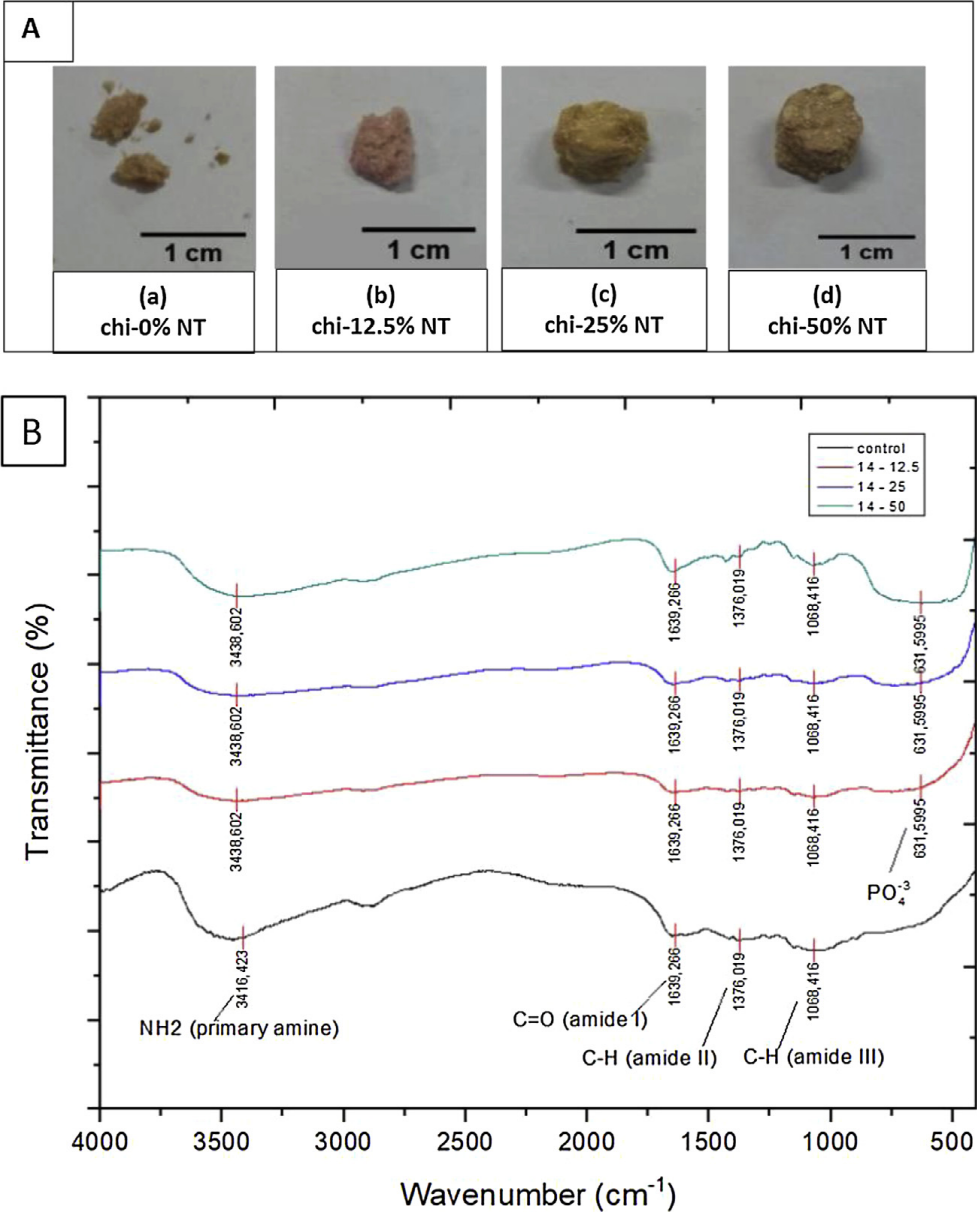
Physical appearance (A) and IR spectrum (B) of samples after incubation in SBF solution.

Fig. 3A showed the physical appearance of sponges after degradation behaviour test using SBF for 2 weeks. Hybridized sponges (in all treatment groups) showed physical integrity up to 2 weeks of incubation in SBF, while control group (chitosan only) was found to be collapsed only within 7 days, as shown in Fig. 3A.

Biomineralization induction was confirmed in this study by FTIR spectroscopy in Fig. 3B. As expected, peaks for C=O amide I (±1600 cm^−1^), C–H amide II (±1300 cm^−1^), C—H amide III (±1000 cm^−1^), and NH_2_ amine (±3400 cm^−1^) that indicate chitosan were found in all samples. Besides, the FTIR of all chi-NT scaffold samples has shown characteristics bands for phosphate groups in ±600 cm^−1^. The carbonate group (±1400 cm^−1^) from bioapatite is overlapped with C—H amide II groups from chitosan, therefore cannot be seen as a clear band.

**Fig. 4:**
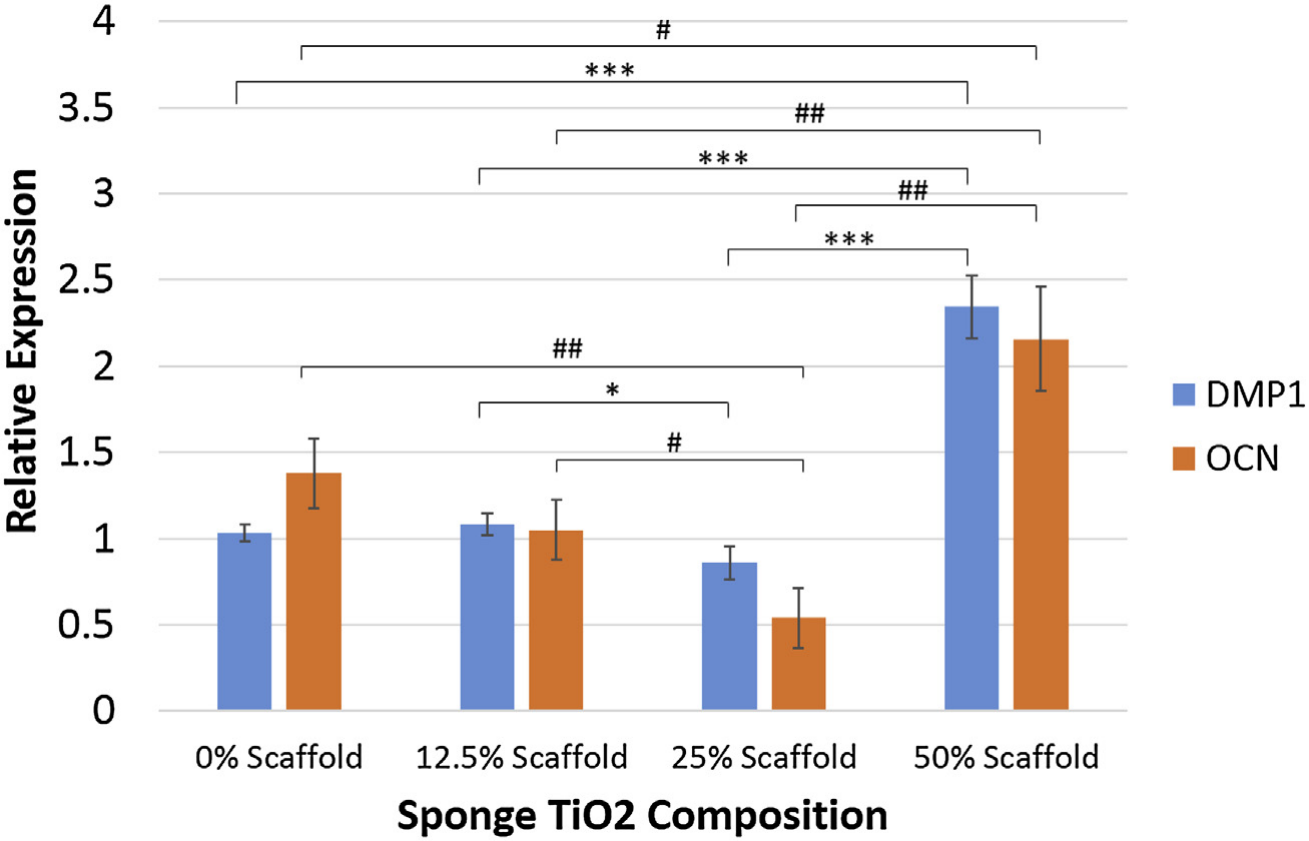
DMP1 gene and OCN gene expressions of cells seeded on the scaffold. An asterisk (*) marks represent significant differences between group for DMP1 gene, and an octothorp (#) marks represent significant differences between group for OCN gene (p-value < 0.05).

Moreover, to strengthen bioactivity potential from scaffold, osteogenic differentiation capability was carried out to investigate the effect of TiO_2_ nanoparticles presence towards bone formation. Fig. 4 shows that addition of 50% TiO_2_ nanoparticles significantly improved bone regeneration capability as shown in DMP1 and OCN gene upregulation more than two-fold compared to control sample (chitosan sponge only).

**Fig. 5:**
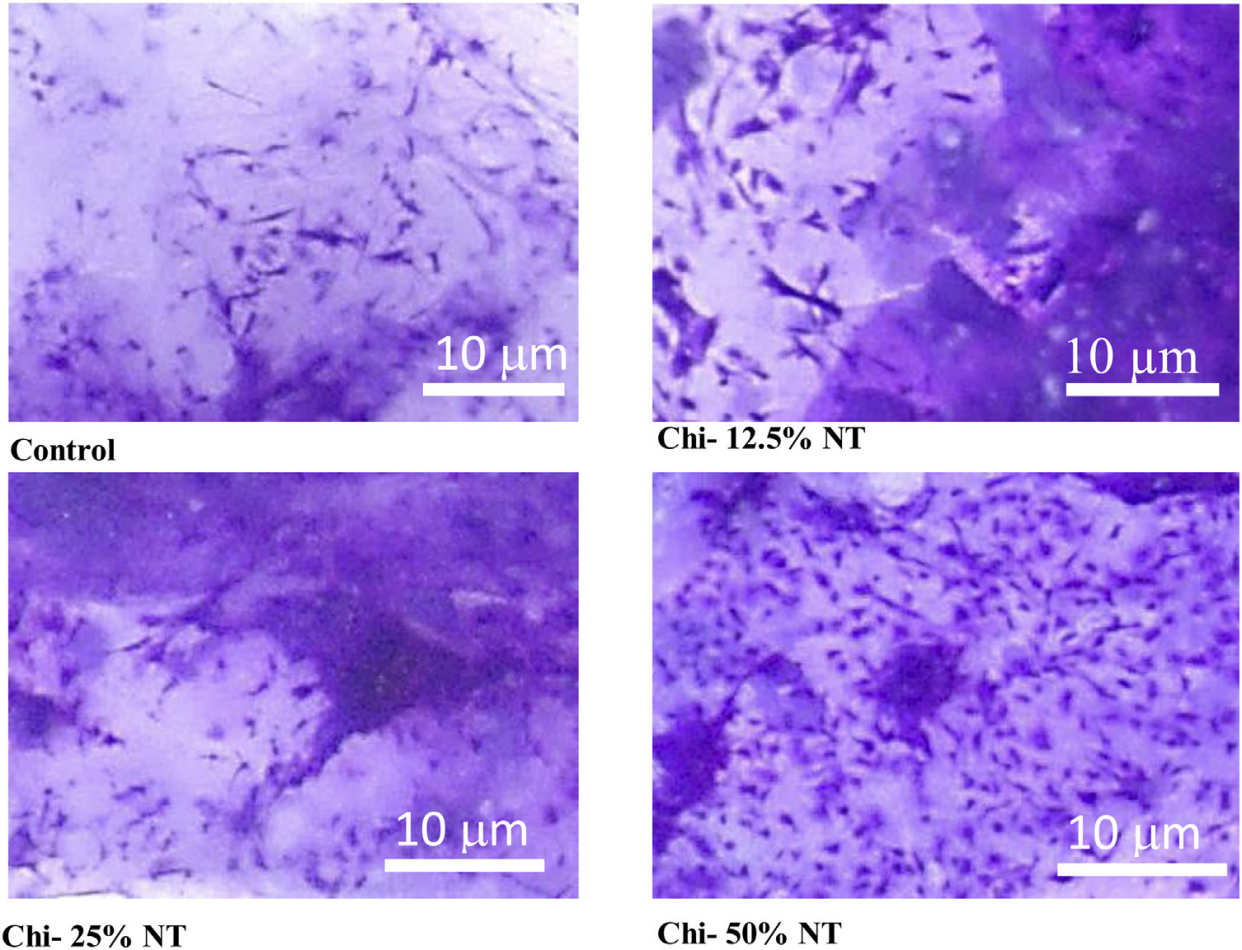
Cell attachment on the scaffold in 40x magnification after staining with crystal violet (Magnification: 1500x).

Biocompatibility was also demonstrated from cell attachment on the sponge and cytotoxicity analysis using WST assay. Fig. 5 showed that higher concentration of nano TiO_2_ led to more cells attached on the scaffold.

**Fig. 6:**
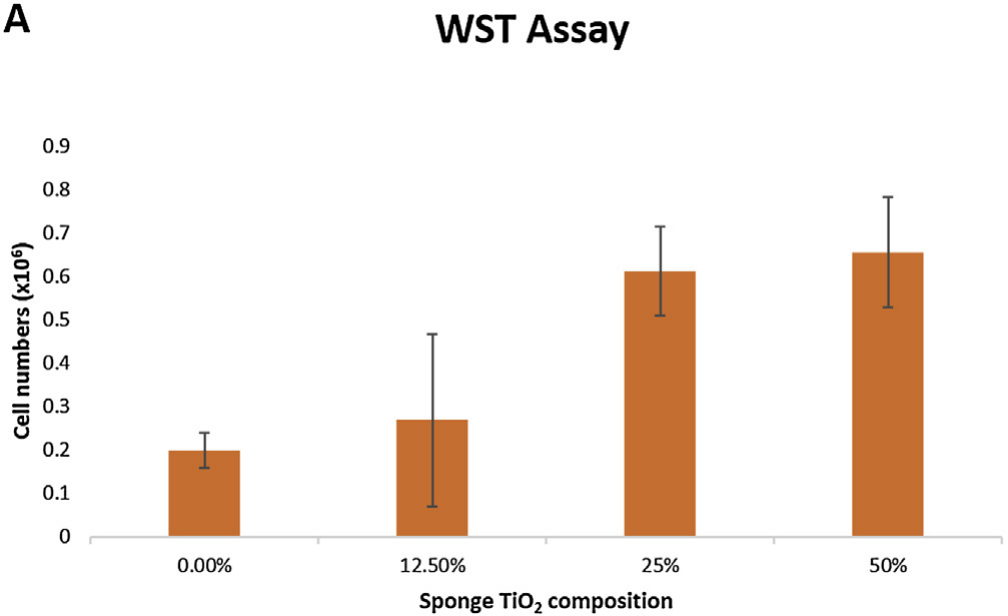
Cytotoxicity analysis using WST assay after 5 days cell culture. Hybridized TiO_2_ on the chitosan scaffold can significantly improve biocompatibility properties of the scaffold.

Cytotoxicity analysis was conducted after 5 days culture and it could be depicted from Fig. 6 that cell could proliferate well in all treatment groups, with a trend of increasing cell number as TiO_2_ concentration increased. These results is in line with the cells attachment image in Fig. 5.

## 4 Discussion

In this work, we investigated the effect of TiO_2_ nanoparticles addition to chitosan sponge, with an idea to improve its mechanical properties and osteoinductive capability. Our results showed that chitosan – nano TiO_2_ sponges could be fabricated in accordance with widely accepted standard of scaffold for tissue regeneration. Scaffolds in tissue regeneration plays a significant role as a site where cells, growth factors and other cytokines to interact, finally to give a shape for tissue regeneration. Scaffolds for tissue regeneration purpose normally need to satisfy following physical characteristics: significant presence of pores, interconnected pores, uniform pore size and even distribution, also robustness (not easily collapsed) [[32], [33], [34]].

We further confirmed that nano TiO_2_ addition to the matrix of chitosan sponges was done successfully, as depicted from an even distribution of nano TiO_2_ on the surface of the sponges. Hybridization of sponges could face several challenges, such as pore collapse and uneven distribution. Pore collapse might happen when new materials addition covered the surface of pores, while uneven distribution could happen due to insufficient reagent or experimental error (insufficient stirring time/speed, incomplete dissolution leading to precipitation, etc.). Even distribution of TiO_2_ nanoparticles on the surface of sponges would ensure that mechanical and biological improvement of the sponges would also be taking place throughout the sponges area. Further existence of nano TiO_2_ on surface of sponges was confirmed by crystallographic analysis using XRD. The data showed that TiO_2_ maintained its crystal structure of anatase following its synthesis process with chitosan sponge. All the data could bring an insight that the hybridization process of chitosan and nano TiO_2_ by means of solvent casting (with aid of lyophilisation) could already deem a proven method. The method was actually a widely-used methods by other researchers to hybridize, for instance, chitosan with biocompatible polymers, such as PLGA, PCL, as well as ceramic materials, such as hyhdroxyapatite, SiO_2_, etc [[35], [36], [37], [38]].

Biological evaluation of sponges also showed promising results. One of the problems faced by chitosan sponges is their relatively fast degradation time. Accordingly, many argued that chitosan is not a suitable candidate for tissue regeneration, especially for hard tissues. In our work, we proved that chitosan with as little as 12.5 wt% addition could maintain its integrity after 2 weeks of culture in simulated body fluid (SBF), while chitosan only sponge already collapsed in day 7. In this study, SBF was also used to evaluate biomineralization from scaffold indicated by apatite formation. SBF has ion concentration equal to human blood plasma. From FTIR results, PO^4−^ band (represents hydroxyapatite formation) was observed in all chitosan–nano TiO_2_ scaffold. Phosphate group is important functional groups indicate hydroxyapatite formation, which indicates its biomineralization induction capability. These results prove that adding TiO_2_ to chitosan scaffold can improve biomineralization activity. Apatite formation has been considered as an important factor for mineralization capability of implants, especially for bone implant [46]. In addition, previous studies [44,45] reported that material can bond to living bone through apatite layer which forms in its surface.

Furthermore, osteogenic induction analysis of the cells seeded on the scaffolds were conducted by examining DMP1 and OCN gene expressions. Dentin Matrix Protein 1 (DMP1) is a bone-specific important marker for osteocyte formation and phosphate metabolism for maintaining mineralization of the bone extracellular matrix. Mutation on DMP1 gene could cause abnormalities in bone development, such as osteomalcia and rickets [47,48]. Whereas, osteocalcin (OCN) plays its role when precursor cells committed to differentiate into mature osteoblasts. Osteocalcin is only secreted by osteoblasts and has endocrine function [49,50]. Both genes are prominent late markers in osteogenic differentiation. According to Fig. 4, sponge with 50% nano TiO_2_ showed the highest expression on DMP1 and OCN gene, indicating sponge ability to support MSCs cells differentiation into osteoblasts. MSCs differentiation into osteoblasts, or osteogenesis is a process that is strongly influenced by the matrix at which differentiation occurs. TiO_2_ improves matrix biomimeticity for bone formation supported by its high bioactivity and higher stiffness/overall mechanical properties that resembles bone in vivo [51]. Nevertheless, addition of nano TiO_2_ up to 25% showed to have reverse effect on the differentiation. Report has showed that nano TiO_2_ presence as a filler have no significant effect for osteogenesis at a considerably low amount [51]. Furthermore, reverse effect on the differentiation might be in effect due to UV irradiation process during sterilization, that causes chitosan to be degraded [52]. Since focal adhesion (FA) is known to be important for the differentiation of osteogenic cells, reduced adhesion due to the degradation of scaffold may suppresses FA formation, stress fiber polarization, cell stiffness and osteogenic commitments in MSCs [53]. Another important issue in this study was the biocompatibility of chitosan-nano TiO_2_ scaffold. The results from microscopic image in Fig. 5 and WST assay in Fig. 6, proved that cells could attach and proliferate well in chitosan – nano TiO_2_ composite sponges. This is in line with the results showing that nano TiO_2_ was considered as a biocompatible material, with no sign of toxicity during culture [[41], [42], [43]]. From these data, chitosan - 50% nano TiO_2_ sponges has demonstrated its prominent ability in biomineralization, osteogenic induction, and biocompatibility.

## 5 Conclusion

Hybridize chitosan with nano TiO_2_ to make a sponge, results in significantly improved sponge robustness, biomineralization and bone regeneration capability, as indicated by DMP1 and OCN gene upregulation in chitosan - 50% nano TiO_2_ group. Therefore, chitosan-50% nano TiO_2_ nanoparticles sponges could be a potential novel scaffold for bone tissue engineering, especially in the cases of complex cavity form.
